# The Combined Effect of Nonalcoholic Fatty Liver Disease and Metabolic Syndrome on Osteoporosis in Postmenopausal Females in Eastern China

**DOI:** 10.1155/2018/2314769

**Published:** 2018-07-29

**Authors:** Da-Zhi Chen, Qiao-Mai Xu, Xiao-Xin Wu, Chao Cai, Ling-Jian Zhang, Ke-Qing Shi, Hong-Ying Shi, Lan-Juan Li

**Affiliations:** ^1^State Key Laboratory for Diagnosis and Treatment of Infectious Diseases, Collaborative Innovation Center for Diagnosis and Treatment of Infectious Diseases, The First Affiliated Hospital, College of Medicine, Zhejiang University, Hangzhou 310003, China; ^2^Department of Infection and Liver Diseases, Liver Research Center, The First Affiliated Hospital of Wenzhou Medical University, No. 2 Fuxue Lane, Wenzhou 325000, China; ^3^Department of Preventive Medicine, School of Public Health and Management, Wenzhou Medical University, Wenzhou 325000, China

## Abstract

The present study evaluated the potential combined effects of NAFLD and MetS on the development of osteoporosis. The relationship between NAFLD and MetS and osteoporosis was assessed in 938 postmenopausal female participants. Moderate and severe NAFLDs were combined as significant NAFLD (SNAFLD). All the subjects were divided into 4 subgroups based on the status of SNAFLD and MetS. Relative excess risk of interaction (RERI), attributable proportion (AP) of interaction, and synergy index (SI) were used to investigate the additive interaction of those two factors. NAFLD, SNAFLD, and MetS were independent factors for osteoporosis with the adjustment of age and other confounders. The incidence of osteoporosis in MetS (+) SNAFLD (+) group was significantly higher than that in other three groups. RERI was 2.556 (95% CI = 0.475–4.636), AP was 0.454 (95% CI = 0.201–0.706), and SI was 2.231 (95% CI = 1.124 to 4.428), indicating the significant combined interaction of SNAFLD and MetS on the development of osteoporosis. SNAFLD and MetS are independent risk factors for osteoporosis in postmenopausal females, respectively. Moreover, SNAFLD and MetS have an additive effect on the development of osteoporosis.

## 1. Introduction

Osteoporosis is defined as a systemic skeletal disease that reduces bone strength and enhances the risk of bone fractures, which result from alteration of bone mass and microstructure. According to the World Health Organization (WHO) definition, osteoporosis is identified as a bone mineral density (BMD) that lies 2.5 standard deviations (SDs) or more below the average for the young healthy adults [1]. It has been anticipated that the osteoporosis population in China will climb dramatically from 83.9 million to 212 million from 1997 to 2050 [2]. Therefore, osteoporosis is an important public health issue in China. In addition, osteoporosis incidence is gender-dependent. Li et al. [3] investigated 5593 Chinese of Han nationality indicated that the total incidence of osteoporosis among people aged over 40 years in 2002 was 16.1%, with the prevalence among males at 11.5% and females at 19.9%.

Osteoporotic fracture is one of the most frequent complications of osteoporosis, which can cause poor life quality and even death [4–7]. Patients with osteoporostic fractures usually require a short- or long-term professional health nursing that increases the economic burden. Reports showed that a long-term stay in skilled nursing facility is needed for approximately 20% of patients experiencing hip fractures, and more than half of those patients cannot recover their prefracture level of functional independence [5]. Furthermore, osteoporotic fracture is considered to be a significant risk factor of mortality. Abrahamsen et al. [8] reviewed related literatures and found that the patients with hip fracture had a 36% excess mortality in the first year compared with adults who did not have hip fracture. Thus, investigating the risk factors for potential osteoporosis and identifying high-risk patients are critical for prevention and treatment of osteoporosis.

Nonalcoholic fatty liver disease (NAFLD), one of the most prevalent chronic liver disorders worldwide [9, 10], is considered to be a risk factor for the development of osteoporosis regardless of genders [11, 12]. On the other hand, MetS, a disorder composed of different metabolic derangements including central obesity, hypertension, dysfunction in glucose, and dyslipidemia, is also proved to increase the risk of developing osteoporosis [12, 13]. There are several studies investigating the association of osteoporosis and NAFLD and MetS, but no one estimated the additive effect of NAFLD and MetS on osteoporosis development. In addition, previous studies indicated that osteoporosis is more frequently observed in females, which supports osteoporosis is gender-dependent [14–19]. Also, osteoporosis is an age-specific disease due to age has huge impact on the hormone secretion and physical status [20]. Thus, our study aimed to investigate the associated factors for osteoporosis in postmenopausal females.

## 2. Patients and Methods

The subjects enrolled in our study from communities accepted routine health status checkup in our hospital from February 2011 to March 2016, and each participant routinely accepted BMD examination. Postmenopausal status was defined as cessation of menses for at least 1 year and serum follicle-stimulating hormone concentration > 30 IU/l. Participants with liver diseases, including HBV, HCV, autoimmune hepatitis, and other chronic liver diseases that can influence liver function, were excluded. Females with a history of alcohol consumption that was more than 20 gram/day alcohol were not considered eligible for the study to exclude the possibility of alcohol-related disease. In addition, participants who met at least one of the following conditions were excluded from the study: had a family history of osteoporosis, an inactive lifestyle, a diet low in calcium or vitamin D and/or high in caffeine, the presence of acute infection or chronic inflammatory disease, and history of early menopause and nulliparity. Participants were also excluded if they were taking medications that could affect metabolism, such as *β*-blockers, glucocorticoids, lipid-lowering agents, antiresorptive agents, diuretics, and anticoagulants.

Finally, the present study involved in 938 postmenopausal female participants, who all came from eastern China. The study was approved by the Ethics Committee of The First Affiliated Hospital of Zhejiang University, and informed consent was obtained from each subject. The study procedure conformed the Helsinki Declaration and strengthening the reporting of observational studies in epidemiology (STROBE) statement [21].

### 2.1. Patients' Basic Characteristics and Laboratory Measurements

A standard questionnaire was applied to obtain the basic information from every subject, including past medical history, current use of medications, alcohol consumption, and family medical. Additionally, participants' height and weight were measured by trained nurses. Subjects were requested to wear lightweight hospital gowns and no shoes when measuring the height and weight. The body mass index (BMI) was calculated as kg/m^2^. Blood pressure (BP) was measured in rest state with a standard mercury sphygmomanometer. Laboratory assays and measurements including fasting glucose, waist circumference (WC), osteocalcin, vitamin D, alkaline phosphatase (ALP), *γ*-acylase, HbA1c, total cholesterol (TC), low-density lipoprotein (LDL) cholesterol, high-density lipoprotein (HDL) cholesterol, triglycerides (TG), aspartate aminotransferase (AST), alanine aminotransferase (ALT), and calcium were performed for all the participants.

### 2.2. Definition of NAFLD and MetS

Hepatic ultrasound scan (Siemens, Germany) was performed by experienced radiologists blinded to the result of osteoporosis for each participant. Participants met specific ultrasonographic features including hepatomegaly, diffusely increased echogenicity of liver parenchyma, and blurring of vasculature were diagnosed as NAFLD [22]. Moreover, NAFLD was further categorized into mild, moderate, and severe level. The detailed diagnosed standard is as follows: patients with slight increase in liver echogenicity, mild attenuation of the penetration of ultrasound signal, and slight decreased lucidity of the borders of intrahepatic vessels walls and diaphragm are identified as mild NAFLD; those with diffuse increase of liver echogenicity, greater attenuation of the penetration of ultrasound signal, decrease of the visualization of intrahepatic vessels walls, particularly the peripheral branches, were defined as moderate NAFLD; and subjects with gross increase of liver echogenicity, greater reduction of penetration of ultrasound signal, and poor or no visualization of intrahepatic vessels walls and diaphragm are considered as severe NAFLD [23]. Then, moderate and severe NAFLDs were considered as significant NAFLD (SNAFLD).

Given the unique feature of Chinese population, we utilized the guidelines proposed by the Diabetes Society of Chinese Medical Association in 2004 for MetS criteria [24]. The MetS was defined as the presence of equal or more than three of the following components: (i) BMI ≥ 25 kg/m^2^; (ii) antihypertensive drug administration and/or systolic blood pressure ≥ 140 mmHg or diastolic blood pressure ≥ 90 mmHg; (iii) TG ≥ 1.7 mmol/l and/or HDL < 0.9 mmol/l (male), <1.0 mmol/l (female); and (iv) fasting plasma glucose ≥ 6.1 mmol/l or 2 h postprandial glucose ≥ 7.8 mmol/l.

### 2.3. Bone Mineral Density Assessment

BMD was detected using the dual-energy X-ray absorptiometry method (Hologic Discovery W series DEXA device, Bedford, MA, USA). DEXA is utilized in our hospital for all the participants due to a low radiation dose, rapid performance, and high accuracy. BMD measurements were obtained for the femur (neck, trochanter, intertrochanteric region, and total), lumbar spine (anteroposterior aspects of L1–L4), and total hip. Osteoporosis is defined as having a *T* score ≤ −2.5 at any of the three tested sites.

### 2.4. Statistical Analysis

The statistical analysis was performed with the SPSS 21.0 (SPSS, Chicago, IL, USA). The statistical results are presented as the mean ± standard deviation or percentages. Independent sample Student's *t*-test was used for continuous variables and chi-square for categorical variables. The relationship of NAFLD, SNAFLD, and MetS with the presence of osteoporosis was assessed by multiple logistic regression analysis after adjustment for confounder variables. And the MetS-related factors including BMI, SBP, DBP, TG, HDL, and fasting glucose were excluded even when they were significant in univariate analysis. Each odds ratio (OR) is presented together with its 95% confidence interval (CI). *P* < 0.05 was considered statistically significant.

Logistic regression analysis was also performed to estimate the probability of the presence of osteoporosis and the 95% CI for each risk factor category stratified by SNAFLD and MetS, adjusting for age and sex. Meanwhile, the relative excess risk (RERI), attributable proportion (AP), and the synergy index (SI) were utilized to evaluate the interactive effect of SNAFLD and MetS on the presence of osteoporosis. The RERI evaluates the excess risk attributed to interaction relative to the risk without exposure. AP is used to measure the attributable proportion of the osteoporosis, which was caused by an interactive effect in patients exposed to those two factors. And SI represents the excess risk from exposure to those two factors when there is a biological interaction relative to the risk from exposure to both without interactive effect. Following the methods proposed by Andersson et al. [25], an interaction was tested on an additive scale by calculating the RERI due to interaction (RERI = RR11 − RR10 − RR01 + 1), the AP due to interaction (AP = RERI/RR11), and the SI [SI = (RR11−1)/(RR01−1) + (RR10−1)]. Both the point estimation and the 95% CI of the RERI, AP, and SI were evaluated applying a method accounting for the asymmetric distribution of confidence limits for risk ratio [26]. When there is no additive interaction, RERI and AP include 0 or S includes 1. And RERI > 0, AP > 0, or S > 1 is considered to have biological interaction [27]. All tests were considered significant when the *P* < 0.05.

## 3. Result

### 3.1. Comparison of Clinicopathological and Laboratory Results for Subjects with or without Osteoporosis

Among the 938 subjects in the present study, 244 subjects (26.0%) were diagnosed as osteoporosis and 232 subjects (24.7%) as MetS. The mean age was 61.0 ± 7.8 years, ranged from 50 to 90 years. Additionally, there were 365 subjects (38.9%) identified as NAFLD with 132 of those NAFLD (14.1%) as SNAFLD. Baseline characteristics of all the participants are summarized in Table 1. The age, serum total cholesterol (TC), osteocalcin, vitamin D, ALP, NAFLD, SNAFLD, and MetS had significantly correlation with osteoporosis. Multivariate analysis with adjustment of age and other significant factors in univariate analysis was applied to evaluate the relationship between osteoporosis and MetS, NAFLD, as well as SNAFLD. We utilized two multivariate analysis models, which are based on the two categories for NAFLD (model 1) and SNAFLD (model 2), respectively. As shown in Table 2, model 1 showed MetS, age, and ALP were independent risk factors for osteoporosis (*P* = 0.002, *P* = 0.019, and *P* = 0.029, resp.), while the presence of NAFLD failed to be significantly correlated with osteoporosis. In model 2, except that MetS and age were still risk factors for the osteoporosis (95% CI 1.319–4.917, OR 2.964, and *P* = 0.005), SNAFLD was significantly correlated with osteoporosis (95% CI 1.389–6.717, OR 3.316, and *P* = 0.001).

### 3.2. Additive Interaction of NAFLD and MetS on Osteoporosis

Next, we grouped patients into 4 groups according to the status of SNAFLD and MetS including MetS (+) SNAFLD (+), MetS (+) SNAFLD (−), MetS (−) SNAFLD (+), and MetS (−) SNAFLD (−) groups, as shown in Table 3. The MetS (+) SNAFLD (+) group has the highest incidence of osteoporosis compared with other subgroups. Table 3 shows that the RERI was 2.556 with 95% CI ranged 0.474 to 4.636, and AP was 0.454 with 95% CI ranged 0.201 to 0.706 for evaluating the additive effect of SNAFLD and MetS on osteoporosis.

## 4. Discussion

The relationship between osteoporosis and NAFLD has been extensively investigated in recent years, but the conclusion is still controversial. Several studies report that NAFLD is a risk factor for osteoporosis. Moon et al. [11] investigated 265 female patients and found that NAFLD was associated with low BMD in postmenopausal women. Cui et al. [28] indicated that patients with NAFLD had significantly lower hip and femoral neck BMD compared with control subjects in both male and postmenopausal female. However, other studies showed opposite results. Xia et al. [29] enrolled 755 men and 1028 postmenopausal women and found that NAFLD was negatively associated with BMDs in men, but no relationship among postmenopausal women. Li et al. [30] found that NAFLD was significantly associated with an increased rate of osteoporotic fractures in middle-aged elderly men, but not in women. Interestingly, Lee et al. [31] showed that the NAFLD had a positive effect on lumbar spine BMD in postmenopausal women.

In the current study, we initially found that both mild NAFLD and moderate/severe NAFLD were associated with osteoporosis, but only moderate/severe NAFLD was the independent risk factor for osteoporosis with adjustment of other confounders. Serum level of ALT is a well-defined biomarker for NAFLD and is useful to predict the degree of steatosis [32]. A study indicated simple steatosis had no influence on BMD, and female NAFLD patients with elevated serum ALT levels had significantly lower BMD [33]. Additionally, NAFLD and elevated ALT can cause a significant synergistic worsening of the BMD in all bone sites [29]. Therefore, we hypothesized that NAFLD is a critical risk factor in the presence of osteoporosis, but is severity-dependent. Thus, the controversial results in the previous studies may be partially explained by the different degree of NAFLD. Our result also showed that only SNAFLD is independent factor for osteoporosis, which can support the above hypothesis, to some extent. Additionally, the diverse dietary habit and heredity of subjects in the previous studies may also lead to the different results.

The MetS consists of at least three components, and each component has intersective and independent mechanisms influencing osteoporosis, which is still unclear. The HDL cholesterol and hypertension are considered to induce osteoporosis [34], while insulin resistance is considered as a protective factor [35]. Additionally, WC shows a negative association with BMD in the previous researches, particularly lumbar spine site [36, 37]. Oppositely, study also demonstrates that WC is not associated with osteoporosis [38], which is similar to our result. The potential explanation is the latter and our studies both defined osteoporosis as *T* score ≤ −2.5 at any site, that is, not only lumbar site. In addition, osteoporosis is a severe condition of low BMD, which may induce the different results.

Totally speaking, the association of MetS and osteoporosis is still inconclusive. Specifically, positive, negative, and not significant relationships between the two diseases were reported previously. Kim et al. [39] indicated that certain components of MetS were negatively associated with BMD in both women and men. Specifically, WC, DBP, and high-density lipoprotein cholesterol were negatively associated with spine BMD in premenopausal women, while only WC was negatively associated with femoral neck BMD in postmenopausal women. Szulc et al. [40] studied 762 older men and indicated that men with MetS had lower BMD. Kim et al. [41] researched a total of 2888 Koreans (1780 men aged 40 years and older and 1108 postmenopausal women) and found that MetS subjects had lower BMD at the femoral neck compared to normal subjects. Hwang and Choi [42] recruited 2548 women subjects aged equal or more than 18 years and found that women with MetS had a lower vertebral BMD. In a recent study conducted by Wang et al. [43] involving 9930 Chinese adults aged 40 years or older in China, it was found that the occurrence of osteoporotic fracture was higher among women with MetS, but not among men. Oppositely, a few studies report a positive or even no relationship between MetS and bone health [44–46]. In our study, we found that MetS was an independent risk factor for osteoporosis by both univariate and multivariate analysis.

To our knowledge, there is no study investigating the additive effect of NAFLD and MetS on osteoporosis before. Recently, it is found that NAFLD can occur independently of insulin resistance and the MetS, and furthermore, there is evidence for not only reciprocal causality between these two diseases but also each acting as a perpetuating or exacerbating factor for the other [47–50]. Thus, NAFLD is not a simple component of MetS. In addition, the synthetic effect of NAFLD and MetS has been found in various diseases [51, 52]. Therefore, we aimed to evaluate the interactive effect of SNAFLD and Mets on osteoporosis in postmenopausal females. All the patients were divided into 4 subgroups based on the status of SNAFLD and MetS. We found that the value of RERI was 2.556 with 95% CI ranged 0.475 to 4.636, which indicated that there was a strong additive interaction between SNAFLD and MetS on osteoporosis, and there would be 2.556 relative excess risk that contributed by the additive interaction. Moreover, AP was 0.454 with 95% CI ranged 0.201 to 0.706, suggesting that 45.4% osteoporosis exposed to the two risk factors was caused by the additive interaction of SNAFLD and MetS. Taken together, the result showed that there was a strong additive interaction of SNAFLD and MetS on the osteoporosis onset, and the increased risk of osteoporosis prompting by the presence of both SNAFLD and MetS was significantly higher than by either SNAFLD or MetS alone. Thus, this significant additive interaction indicates that MetS might attribute an extra risk of osteoporosis for patients who also have SNAFLD.

The pathophysiological links between NAFLD and low BMD are still not completely understood. NAFLD can affect osteoporosis through multiple mechanisms, and inflammation plays a crucial role among those mechanisms and may cause osteoporosis via both genetic and acquired factors [53–57]. It is known that MetS also can cause systemic inflammation, which could affect bone formation [58]. In addition, NAFLD encompasses a histological process that starts from a simple steatosis, which only has fat accumulation in hepatocytes but without inflammation, to moderate and severe forms of NAFLD such as steatohepatitis, a condition that hepatic steatosis accompanies with a necroinflammatory component. Thus, the moderate or severe NAFLD may be more strongly associated with osteoporosis onset and development. Furthermore, there is likely an inflammation-mediated crosstalk between SNAFLD and MetS on osteoporosis.

To our knowledge, this is the first study to evaluate the synergetic effects of SNAFLD and MetS on osteoporosis. The present study has several limitations. Firstly, the cross-sectional study design may not provide evidence to explain the potential mechanism of the combined interaction of SNAFLD and MetS on the presence of osteoporosis. Secondly, the present study was a retrospective study, which may have selection bias. Then, SNAFLD in the study was diagnosed by ultrasound imaging examination that is the most prevalent method of diagnosing NAFLD in clinical practice with acceptable high sensitivity (89%) and specificity (93%) [59]. And this study did not provide bone metabolic markers, for example, serum carboxy-terminal collagen cross-links (CTX) for each patient. In addition, the study was conducted on a single center. Thus, the results obtained in the current study need to be evaluated by further multiple-center studies with larger sample size.

## 5. Conclusions

The present study suggests that SNAFLD and MetS are both independent risk factors for osteoporosis in postmenopausal females. MetS and SNAFLD may play an additive effect to the development of osteoporosis in postmenopausal females. Thus, postmenopausal women with both SNAFLD and MetS are recommended to be monitored for the BMD. Also, the potential mechanism of the combined effects of SNAFLD and MetS should be investigated in the future.

## Figures and Tables

**Table 1:** Univariate analyses of associations between osteoporosis and clinical features in postmenopausal females.

Characteristics	Total number	With osteoporosis	Without osteoporosis	*P* value
	(*n* = 938)	(*n* = 244)	(*n* = 694)	
Age (years)	61.21 ± 13.81	64.13 ± 8.21	58.81 ± 12.31	0.021^∗^
BMI (kg/m^2^)	23.41 ± 3.34	23.13 ± 3.12	24.73 ± 4.17	0.107
SBP (mmHg)	144.71 ± 23.3	147.55 ± 23.72	143.32 ± 21.21	0.131
DBP (mmHg)	72.31 ± 12.11	68.21 ± 8.31	73.01 ± 11.17	0.231
TC (mmol/l)	4.81 ± 1.21	5.18 ± 1.01	4.72 ± 1.31	0.022^∗^
TG (mmol/l)	1.81 ± 1.56	1.93 ± 1.82	1.78 ± 1.63	0.679
HDL (mmol/l)	1.59 ± 0.51	1.86 ± 0.33	1.36 ± 1.33	0.095
LDL (mmol/l)	2.75 ± 0.92	2.81 ± 0.91	2.69 ± 1.05	0.382
Fasting glucose (mmol/l)	6.16 ± 2.81	6.38 ± 3.02	6.08 ± 2.92	0.108
Waist circumference (cm)	104.2 ± 9.36	103.8 ± 8.91	103.5 ± 9.68	0.6885
Osteocalcin (ng/ml)	18.56 ± 4.47	21.68 ± 5.02	17.24 ± 3.82	<0.001^∗^
Vitamin D (ng/dl)	14.76 ± 1.98	16.72 ± 1.52	13.96 ± 1.49	<0.001^∗^
ALP	77.66 ± 43.21	84.22 ± 53.24	75.16 ± 35.12	0.002^∗^
*γ*-Acylase	43.15 ± 91.62	48.63 ± 81.41	42.27 ± 64.18	0.452
HbA1c	10.15 ± 2.10	9.91 ± 1.82	10.20 ± 2.22	0.372
AST (U/l)	28.3 ± 9.2	29.1 ± 10.2	27.6 ± 11.1	0.171
ALT (U/l)	29.8 ± 16.2	31.5 ± 15.1	29.2 ± 17.1	0.091
Calcium (mg/dl)	9.32 ± 0.48	9.25 ± 0.57	9.33 ± 0.52	0.321
Total hip BMD (gm/cm^2^)	0.962 ± 0.098	0.762 ± 0.121	0.981 ± 0.110	<0.001^∗^
Femoral neck BMD (gm/cm^2^)	0.789 ± 0.101	0.701 ± 0.091	0.817 ± 0.125	<0.001^∗^
Lumbar spine BMD (gm/cm^2^)	0.982 ± 0.141	0.843 ± 0.131	1.091 ± 0.113	<0.001^∗^
Total hip *T* score	−0.687 ± 0.865	−1.801 ± 0.891	−0.231 ± 0.981	<0.001^∗^
Femoral neck *T* score	−1.512 ± 0.878	−2.500 ± 0.718	−1.381 ± 0.981	<0.001^∗^
Lumbar spine *T* score	0.102 ± 1.231	−2.351 ± 1.018	0.376 ± 1.109	<0.001^∗^
NAFLD	365 (38.9)	116 (47.5)	249 (35.9)	0.002^∗^
SNAFLD	132 (14.1)	64 (26.2)	68 (9.7)	<0.001^∗^
MetS	232 (24.7)	86 (35.2)	146 (21.0)	<0.001^∗^

NAFLD: nonalcoholic fatty liver disease; SNAFLD: significant nonalcoholic fatty liver disease; MetS: metabolic syndrome; BMI: body mass index; SBP: systolic blood pressure; DBP: diastolic blood pressure; TC: serum total cholesterol: TG: triglyceride; HDL: high-density lipoprotein; LDL: low-density lipoprotein; AST: aspartate aminotransferase; ALT: alanine aminotransferase; ALP: alkaline phosphatase; BMD: bone mineral density; HbA1c: glycosylated hemoglobin. Note: ∗ represents *P* value < 0.05.

**Table 2:** Multivariate analyses of associations between osteoporosis and clinical features in postmenopausal females.

Characteristics	Model 1		Model 2	
	Osteoporosis (+) versus osteoporosis (−)		Osteoporosis (+) versus osteoporosis (−)	
	OR (95% CI)	*P* value	OR (95% CI)	*P* value
Age (years)	1.241 (1.021–1.347)	0.019^∗^	1.114 (1.113–1.441)	0.015^∗^
TC (mmol/l)	1.314 (0.983–1.572)	0.291	1.243 (0.919–1.472)	0.324
ALP	1.104 (1.031–1.447)	0.029^∗^	1.311 (0.891–1.532)	0.112
NAFLD	1.664 (0.989–3.117)	0.121		
SNAFLD			3.316 (1.389–6.717)	0.001^∗^
MetS	3.164 (1.281–5.213)	0.002^∗^	2.964 (1.319–4.917)	0.005^∗^

TC: serum total cholesterol; ALP: alkaline phosphatase; NAFLD: nonalcoholic fatty liver disease; SNAFLD: significant nonalcoholic fatty liver disease; MetS: metabolic syndrome; OR: odds ratio; CI: confidence interval. Note: ∗ represents *P* value < 0.05.

**Table 3:** Measures for estimation of biological interaction between MetS and SNAFLD for the risk of osteoporosis in postmenopausal females.

	Total number	Total case	*P* value	OR (95% CI)	*P* value
MetS (−) SNAFLD (−)	641	126 (19.7)	<0.001^a^	1.0	
MetS (−) SNAFLD (+)	64	24 (37.5)	0.023^b^	2.132 (1.166–3.900)	0.011^∗^
MetS (+) SNAFLD (−)	164	54 (34.1)	<0.001^c^	1.945 (1.162–3.253)	0.031^∗^
MetS (+) SNAFLD (+)	68	40 (58.8)		5.632 (3.281–9.666)	0.001^∗^
Measures of biological interaction		Estimate (95% CI)			
RERI		2.556 (0.475–4.636)			
AP		0.454 (0.201–0.706)			
SI		2.231 (1.124–4.428)			

SNAFLD: significant nonalcoholic fatty liver disease; MetS: metabolic syndrome. Note: data are expressed as *n* (%); ∗ represents the *P* value ≤ 0.05. ^a^*P* value represents MetS (+) SNAFLD (+) versus MetS (−) SNAFLD (−); ^b^*P* value represents MetS (+) SNAFLD (+) versus MetS (−) SNAFLD (+); ^c^*P* value represents MetS (+) SNAFLD (+) versus MetS (+) SNAFLD (−).

## Abbreviations

NAFLD: Nonalcoholic fatty liver disease
MetS: Metabolic syndrome
BMI: Body mass index
BMD: Bone mineral density
BP: Blood pressure
LDL: Low-density lipoprotein
HDL: High-density lipoprotein
AST: Aspartate aminotransferase
ALT: Alanine aminotransferase
TC: Serum total cholesterol
TG: Triglyceride
RERI: Relative excess risk
AP: Attributable proportion
SI: Synergy index.
